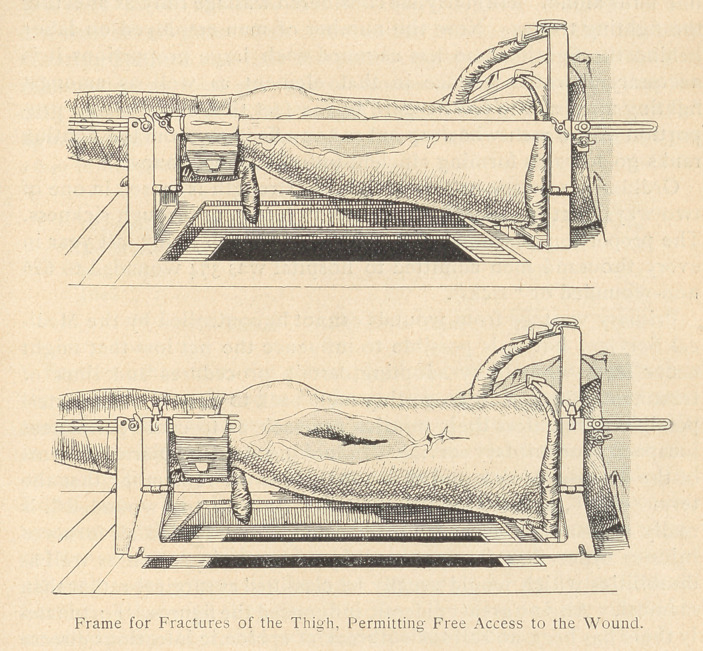# Treatment of Fractures of the Femur

**Published:** 1918-09

**Authors:** 


**Treatment of Fractures of the Thigh.** By Médecin-Major Heitz-Boyer. The speaker limited his remarks on fractures of the thigh to five or six special problems in which he had been able to affect some improvement over previous methods of procedure.

*Evacuation Preceding Hospitalization.* For the immediate transport of fracture cases 30 or 40 kilometers behind the lines, an apparatus affording complete immobilization is essential. The apparatus invented in the service of Heitz-Boyer by Pouliquen affords the necessary immobilization to the maximum degree. The member is supported by a grooved splint, completed by a wooden frame, the outer end of which extends to the armpit.

Pouliquen Splint and Frame.

Extension is performed on the foot by a rubber band; and counter-extension, by a supple band passed obliquely over the groin and fixed under the armpit. This apparatus is the only one which immobilizes the two articulations above and below the fracture, immobilization of the hip being essential in cases of high fractures of the femur. Moreover, it is made of wood, is inexpensive, easily constructed, and may even be improvized anywhere.

*The Removal of Splinters.* This important point in operative surgery has given rise to a divergence of opinion among French surgeons, but Heitz-Boyer thinks that now the solution to the problem has been found.

The removal of splinters where the fracture of the femur has been large ought to fulfil a double mission : — (1) To stop or hinder infection at the start. (2) Later, to foster bony regeneration. The dispute is as to whether, in order to accomplish regeneration, it is necessary to perfom primary preventive ablation of the adherent fragments (for all are agreed that loose splinters should be removed) or, on the contrary, whether only a limited resection be made during the first hours after the injury with a view to making a more extensive secondary resection. There is a further dispute, as to whether primary or secondary ablation affords the best prognosis for complete bone regeneration?

A. With regard to *disinfection*, all are agreed that with the means now available for surgical disinfection, the seat of a fracture can be disinfected without extensive bony damage; just enough bone ought to be removed to ensure good drainage. On the contrary, the soft parts must be very largely excised and carefully flattened after being thoroughly scraped. As for the spine, Heitz-Boyer maintains that from the anatomical and bacteriological point of view, it is untouched, even as far as the confines of the fracture itself; experience having taught him that contusion and infection of the medullary tissue are usually limited to the first centimeter or centimeter and a half above the fracture. It is therefore useless to perform curettage of the spine.

In order to ensure post-operative disinfection of a fracture, Heitz-Boyer prefers, during the first days, the use of ether-alcohol-iodoform which, thanks to an injection renewed morning and evening, permits of the dressing's being left in place for four or five days. The Carrel treatment is then employed, with its daily dressings and non-traumatizing methods.

B. With regard to *bony regeneration*, this, of course, can only take place if the surgeon has left in the wound the bone generating elements. In France it was thought until recently, according to the works of Ollier, that the periosteum was such an element.

According to this belief large bone resections ought to be performed sub-periosteally; that is why Leriche, at the beginning of the war, advocated extensive primary sub-periosteal removal of splinters. But Ollier himself had observed that when this was performed a few hours after wounding, although in a child it would be followed by bony consolidation, in the adult the result was negative. He therefore came to the conclusion that the normal condition of the adult periosteum was one of sterility, and that it did not become fertile until inflamed, i. e., on the 4th or 5th day after an open fracture. Hence the necessity, according to Ollier, of waiting for the "secondary" period before performing extensive subperiosteal resection.

Fig. 1. — Periosteum of Young Bone in Process of Growth after Sub-periosteal Rugination.

Surgically speaking Ollier was right, as the numerous pseudarthroses which have taken place as a result of the technic previously recommended by Leriche. The theoretical explanation, however, which Ollier gave was wrong, for in the speaker's opinion, at no time of life does the periosteum generate bone. " *Only bone itself generates bone* ". This theory, when defended last year by Heitz-Boyer together with Scheikewitch before the Académie des Sciences, gave rise to violent criticism in France. It ought not, however, to surprise American surgeons, who, for several years, have raised doubts concerning the osteogenetical properties of the periosteum. If once this conception is admitted, the whole question of esquillectomy and of resection in general, is greatly clarified. The three accompanying diagrams show, indeed, the result of a subperiosteal resection : (1) upon a young bone during the process of growth; (2) upon a matured bone without inflammation; and (3) upon a matured bone in a condition of inflammation on the 4th or 5th day after injury. *In only one of these three cases, that of the normal matured bone* (see fig. 2), *the resection was genuinely subperiosteal. In the two other cases* (see fig. 1 and 3) *the rugine passed through the bone itself.* It could not have been otherwise, because of a

Fig. 2. — Periosteum of Normal Matured Bone after Sub-periosteal Rugination.

very important anatomical fact, the significance of which seems to have been neglected and upon which Heitz-Boyer insists. Indeed, on the young normal bone, as on the inflamed matured bone, the exterior surface is not smooth as in the case of an adult bone; neither has it the hardness peculiar to bone. It is very irregular, and presents a quantity of fragile and brittle granulations which invade the adjacent periosteum and tend to push it back (see fig. 1 and 3). In the case of young bone this anatomical fact is set forth in all books on histology. On a matured inflamed bone, as is the case in a war wound of only a few days standing, the histological examinations of Heitz-Boyer and Sheikewitch have proved, after the fourth or fifth day of a wound, that the injured and inflamed bone presents a double series of lesions of *rarifying* ostitis (see color plates) in the bone itself, with trabeculae and softening of the fundamental tissue, *proliferating* ostitis growing outside the bone itself *irritating* and constituting a genuine " inflammatory bony neoplasia " which invades the periosteum and quickly creates an embryonic medullar bone layer which is connected with the old bone by thin bony attachments. It is at the level of these that the rugine should pass in the process of subperiosteal resection — that is, not at the point where the bone and the periosteum unite, but *at the junction of the old bone and the new*, which has, as it were, forced back the periosteum. Therefore on a young growing bone as upon a matured inflamed bone, a layer of newly formed bone (either physiological growth or pathological reparative bone) should always be removed with the periosteum: Ollier's technique in similar cases is, strictly speaking, bone resection. On the contrary, in the case of matured bone without inflammation (as in primary traumatic esquillectomy), the surface of the bone upon which the rugine has to act is hard and smooth for there is no proliferating or rarifying ostitis; the rugine must then pass between the bone and the periosteum : the resection is then properly speaking subperiosteal.

Fig. 3. — Periosteum of a Mature Bone Inflamed (at the Level of a Fracture, the 4th or 5th Day after Injury) Following Sub-periosteal Rugination.

From the point of view of future bone regeneration, Ollier perfectly understood what was taking place in the three instances mentioned : after the subperiosteal use of the rugine on a healthy bone, no bone growth occurs, whereas in the other two cases, that of the young growing bone and that of the matured inflamed bone, bone regeneration takes place.

*Surgical conclusions* : If regeneration is required, *all esquillectomy or traumatic resection must consist " in cutting the bone itself "*. In the case of mature bone in fractures of five days' standing, that is in cases of primary traumatic esquillectomy, the classic technic must be modified, or " exaggerated ", as Leriche has recently said, by forcible penetration into the hard cortex of the bone, cutting away with a well sharpened rugine, repeatedly renewed, a series of superficial bony parings, in such a manner as to change the condition represented in figure 2 into that shown in figure 4, or, in other words, to transform a subperiosteal resection into a genuine bone resection, which may then be followed by bone regeneration.

Fig. 4. — Periosteum of an Uninflamed Bone after Rugination of the Bone Itself.

In the light of these conceptions, Heitz-Boyer reaches the following conclusions on the subject of esquillectomy in fractures of the femur. A primary esquillectomy may suffice to insure disinfection, although it is limited in extent ; even if a large resection is required, it will always be strictly in the form of bone resection according to the technic of Leriche. A secondary esquillectomy must always be extensive, and is, of course, bone resection, even when the so-called classic subperiosteal technic is employed. At the time of operation, the choice as to whether esquillectomy should be primary or secondary, extensive or limited, will be determined by the *time* and *place* of the operation. (a) In times of moderate activity, if the necessary equipment is at hand, and there is a sufficient number of available rugines, and if the operator is familiar with the new Leriche technic, he will find primary bone resection the procedure of choice, because it suppresses infection more certainly than any other technique, while at the same time it insures the renewal of the bone substance, even after extended bone destruction; in short, it permits post-operative care by simpler and more rapid methods. (b) During times of great activity, on the contrary, or when the equipment is insufficient, or if the operator is not familiar with the Leriche technic, primary esquillectomy should be attempted, if at all, on a very small scale: such treatment will often prevent serious infection and may be supplemented a few days later by a large secondary esquillectomy.

*Immediate or Secondary Closure.*

Heitz-Boyer has succeeded in the practice of primary suture for fractures of the femur; but he sees in this procedure no great advantage over secondary or "delayed primary suture" (Pierre Duval), which leads to the same results a few days later, but with much more certainty.

*Instrumental Technic.*

Heitz-Boyer has been led to introduce a wholly new set of instruments for the treatment of fractures, especially of the femur, for it is frequently necessary, in a large swollen thigh, to penetrate to the depth of 10 or 15 centimeters in order to reach the two diaphyseal fragments and the intervening splinters which constitute a serious danger to the large vessels. This set of instruments consists of:

Spreaders with a spring attachment.

Splinter forceps which clasp the splinter firmly and hold it automatically.

A rugine with a curved handle, adapted to the concavity of the hand.

Daviers of a new model, intended to make the instrument less awkward in the wound. This change consists in an arrangement whereby one of the handles slips inside of the other, in such a manner as to facilitate the closing of the forceps with the help of

Davier with Parallel Grip Responding Instantly to Pressure.

a thumb screw, thus causing a minimum of inconvenience and loss of time. In this manner the Davier is suited to all conditions of the wound and gains easy access to the seat of the bone injury. It is so arranged as to provide any manner of grip, or to permit the male portion of the grip to be used as a crochet for searching out fragments in the wound.

A *guide-réducteur*, to render the fixation of the reduction, and fixation of fragments easier and almost automatic. This appliance is inserted between the Daviers when each is attached to one of the bone fragments. The *guide-réducteur* is composed of two parts: (a) a central and transverse portion constituting a point of leverage between the two Daviers, and transforming each of them into a secondary lever (in this manner the surgeon finds his strength infin-

Showing How the Davier Adapts Itself to Bones of all Shapes and Sizes.

itely increased); (b) two lateral parts, branching off at right angles from the preceding, and consequently parallel to each other. It will be sufficient to bring each of the Daviers into contact with one of the two parallel branches, in order that the two fragments seized by the Daviers may be properly reduced end to end; this result is brought about automatically and almost mathematically[1]. The fragments may be fixed in this position by locking the Daviers upon the lateral branches of the *guide-reducteur*. This manner of fixation at some distance from the seat of the fracture, thus left completely exposed, permits of the practice of every kind of osteosynthesis.

*Equipment.*

The suspension appliances contrived by Blake are excellent :

1. This automatic reduction takes place in accord with the mathematical principle that lines which are perpendicular to parallel lines are parallel to each other.

however, for treatment in advanced hospitals and in the treatment of very grave fractures of the thigh, Heitz-Boyer employs a frame of his own with the Delbet ischiatic and trochanteric points of rest. This frame opens somewhat in the manner of a compass, and makes it possible in three minutes, after the operation has once been completed, to adjust the appliance to the thigh on the operation table itself. On a level with the thigh the lateral branches

Guide-Réducteur Being Introduced between the two Daviers.

may be opened out on both sides in such a manner as to make the wound completely accessible: this arrangement is helpful in dressing, in maintaining asepsis, and in radioscopic investigation. By a special arrangement at the end of the frame, weight extension may quickly be exchanged for spring extension, thus allowing the patient to be transported without the least change in the extension.

*Late Bone Deformities.*

Heitz-Boyer called attention to a late complication in the case of serious bone damage of the femur, which has not previously been reported. When several centimeters of bone growth have taken place and appear to be consolidated, in the third, or sometimes as late as the fifth, month, marked deformities are to be observed. These are to be explained by the evolution of the osteogenetic processes which, as Heitz-Boyer and Sheikewitch have shown, pass through four successive stages : " pre-ossification ", medullar ossification, Haversian changes, and final development. While the

Frame for Fractures of the Thigh, Permitting Free Access to the Wound.

bone is in a condition preceding that of the Haversian changes, it has not as yet reached a stage of consolidation, and may foreshorten or be bent. Unfortunately it is not possible to know the precise moment when the Haversian changes take place, and hence it is necessary to observe most carefully all fractures of the femur for four or five months after the injury.

## Figures and Tables

**Figure f1:**
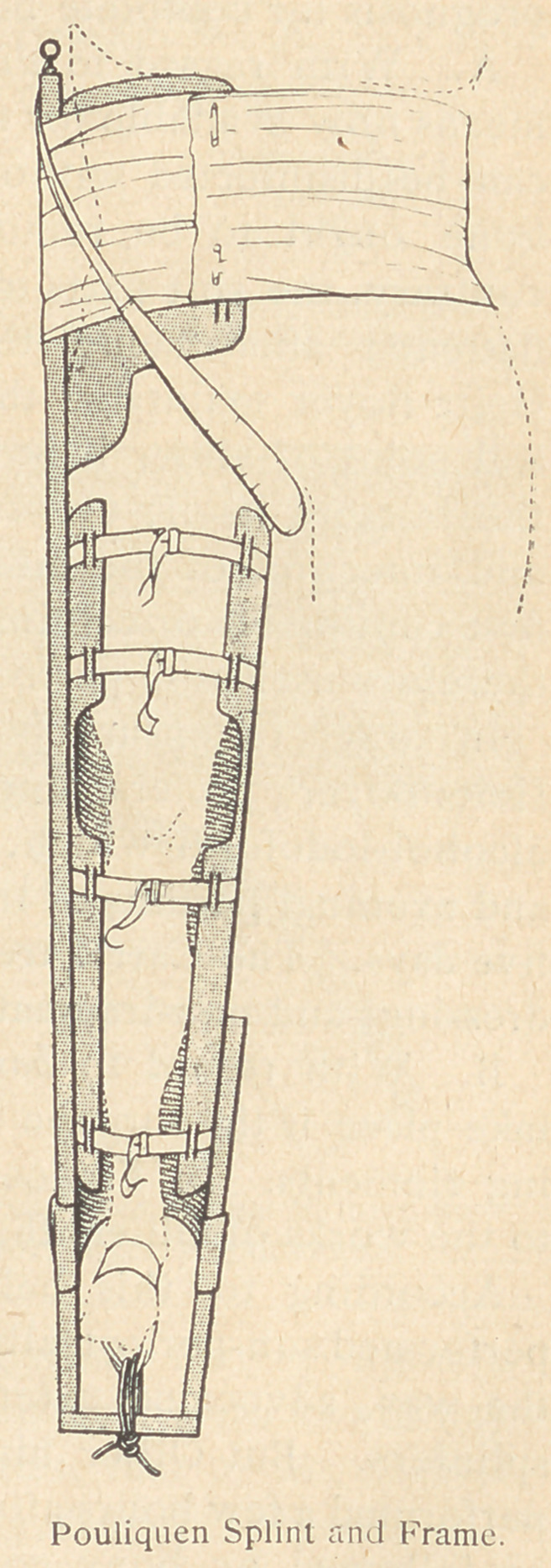


**Fig. 1. f2:**
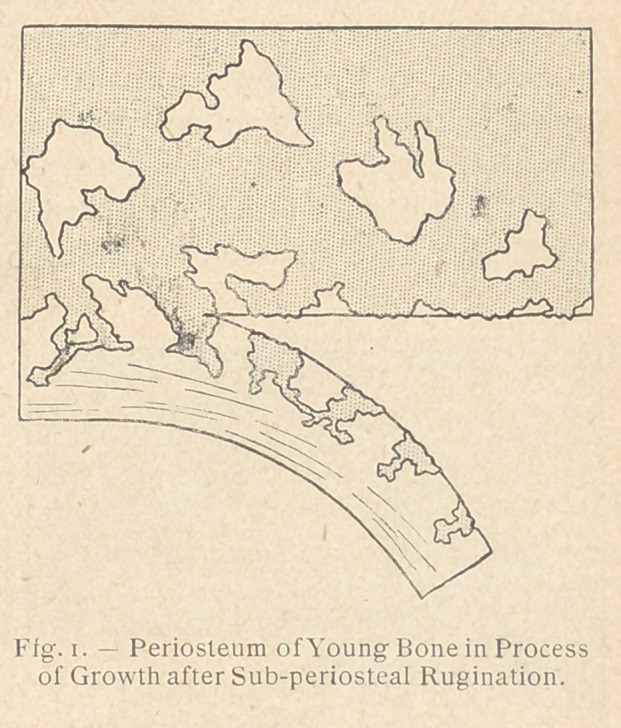


**Fig. 2. f3:**
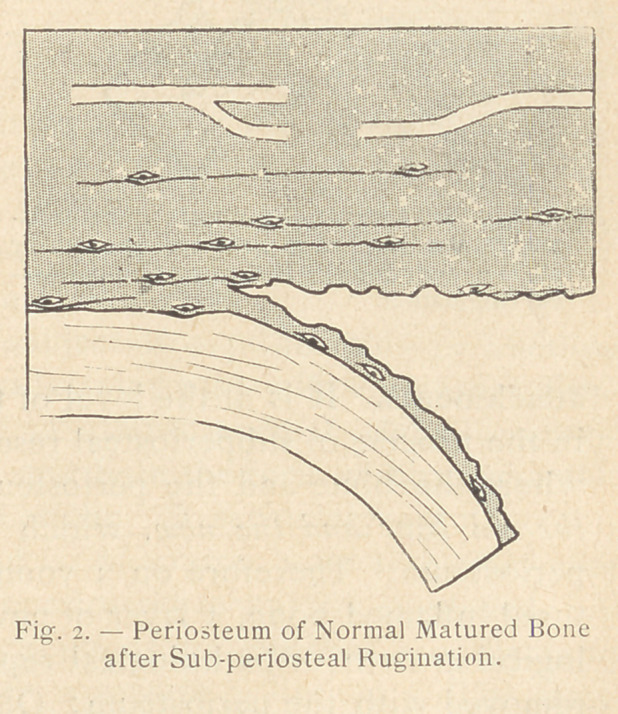


**Fig. 3. f4:**
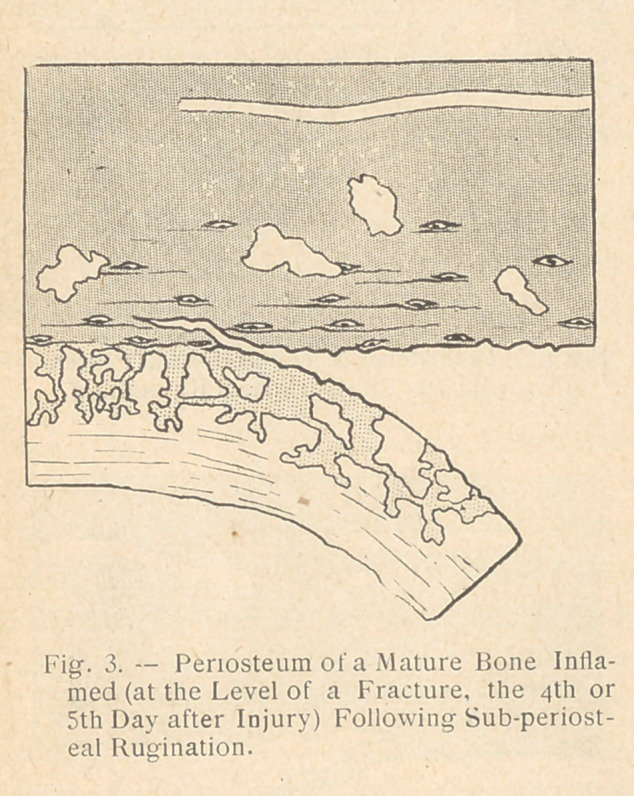


**Fig. 4. f5:**
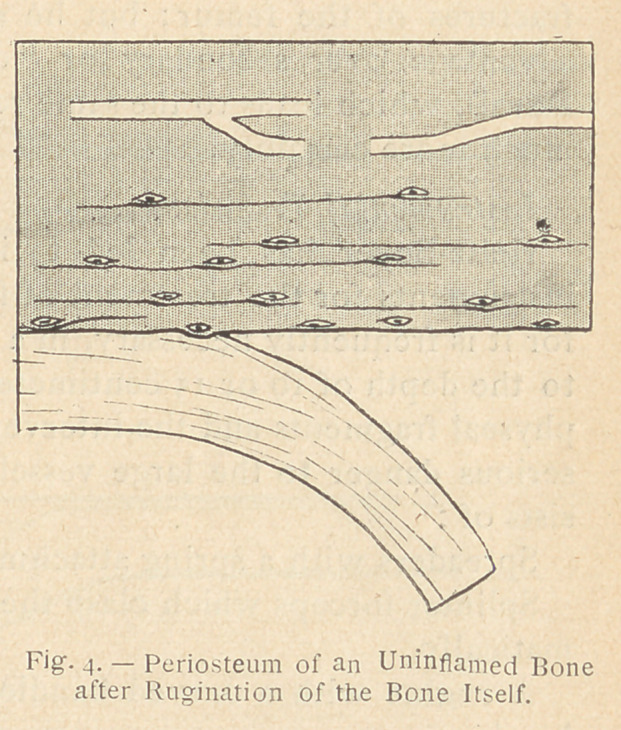


**Figure f6:**
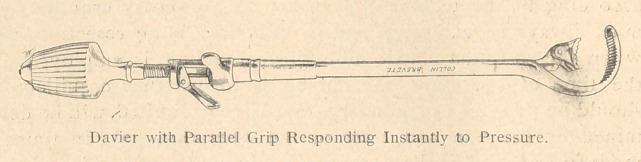


**Figure f7:**
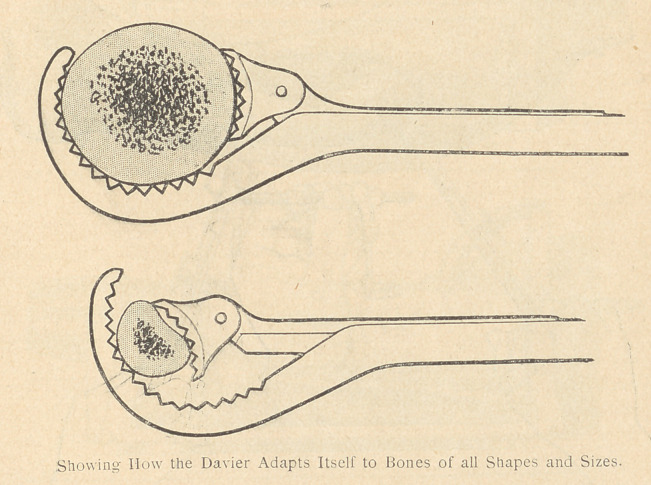


**Figure f8:**
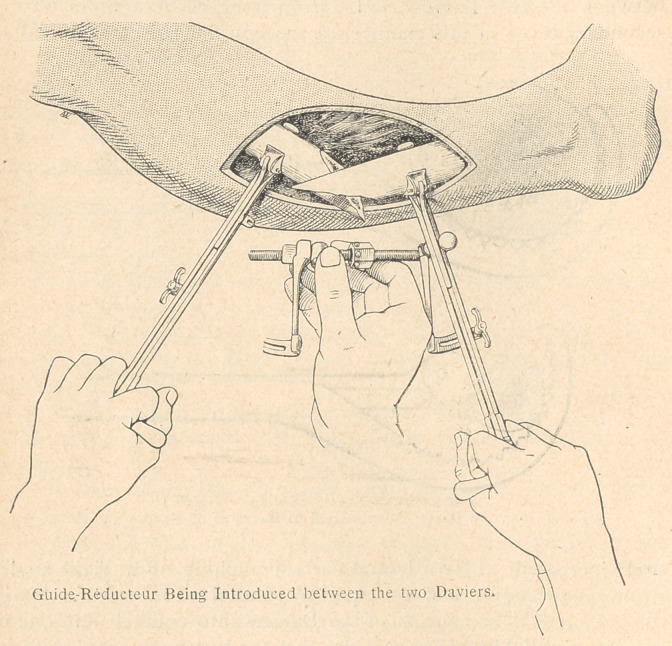


**Figure f9:**